# Assessment of the Incremental Benefit of Computer-Aided Detection (CAD) for Interpretation of CT Colonography by Experienced and Inexperienced Readers

**DOI:** 10.1371/journal.pone.0136624

**Published:** 2015-09-10

**Authors:** Darren Boone, Susan Mallett, Justine McQuillan, Stuart A. Taylor, Douglas G. Altman, Steve Halligan

**Affiliations:** 1 Centre for Medical Imaging, University College London, London, United Kingdom; 2 School of Health and Population Sciences, University of Birmingham, Birmingham, United Kingdom; 3 Centre for Statistics in Medicine, University of Oxford, Oxford, United Kingdom; Sapporo Medical University, JAPAN

## Abstract

**Objectives:**

To quantify the incremental benefit of computer-assisted-detection (CAD) for polyps, for inexperienced readers versus experienced readers of CT colonography.

**Methods:**

10 inexperienced and 16 experienced radiologists interpreted 102 colonography studies unassisted and with CAD utilised in a concurrent paradigm. They indicated any polyps detected on a study sheet. Readers’ interpretations were compared against a ground-truth reference standard: 46 studies were normal and 56 had at least one polyp (132 polyps in total). The primary study outcome was the difference in CAD net benefit (a combination of change in sensitivity and change in specificity with CAD, weighted towards sensitivity) for detection of patients with polyps.

**Results:**

Inexperienced readers’ per-patient sensitivity rose from 39.1% to 53.2% with CAD and specificity fell from 94.1% to 88.0%, both statistically significant. Experienced readers’ sensitivity rose from 57.5% to 62.1% and specificity fell from 91.0% to 88.3%, both non-significant. Net benefit with CAD assistance was significant for inexperienced readers but not for experienced readers: 11.2% (95%CI 3.1% to 18.9%) versus 3.2% (95%CI -1.9% to 8.3%) respectively.

**Conclusions:**

Concurrent CAD resulted in a significant net benefit when used by inexperienced readers to identify patients with polyps by CT colonography. The net benefit was nearly four times the magnitude of that observed for experienced readers. Experienced readers did not benefit significantly from concurrent CAD.

## Introduction

CT colonography (CTC) images the colorectum in both symptomatic [1, 2] and asymptomatic [3, 4] patients. The colon is tubular, long and tortuous, with pathology (sometimes diminutive) occurring anywhere along its length. Additionally, medical image display for CTC is complex, involving both 2D and 3D displays. Accordingly, interpretation is more difficult and time-consuming for radiologists than standard abdominopelvic CT; even highly experienced practitioners may fail aptitude tests [4]. Computer-aided-detection (CAD) aims to improve radiologists’ diagnostic performance by using visual prompts to alert them to pathology that might otherwise be missed [5–7]. CAD prompts may be true- or false-positive.

While it is suggested that CAD may diminish the need for prior experience [8], at the time of writing the two largest published studies have used experienced radiologists alone; 19 [9] and 16 readers [10]. Few studies have compared experienced and inexperienced readers directly, and those that have are limited by their small size and low statistical power [11]. For example, Mang and colleagues asked two “expert” and two “nonexpert” observers to interpret CTC from 52 patients using second-read CAD, finding that CAD was beneficial only for the less experienced readers [6]. The same group also compared concurrent and second-read CAD paradigms, but in “moderately” experienced radiologists alone [12]. A subsequent study by Petrick and co-workers also investigated the second-read paradigm using four readers, two of whom were experienced [13]. Accordingly, the present study aimed to quantify the incremental benefit of CAD for inexperienced versus experienced readers by comparing data across two large multi-reader, multi-case studies of CTC.

## Materials and Methods

### Ethical statement data sources and readers

We obtained individual reader data from two prospective multi-reader, multi-case studies of CAD for CTC [8, 10]. Both had full ethics committee approval for data sharing. All participants had given full informed written consent for data-sharing, and studies were anonymized and allocated a study number by a data manager before being viewed by study readers. Specifically, the research used stored CT colonography images re-interpreted outside of the clinical pathway; patient diagnosis and treatment was not affected by radiological re-interpretation of patient images. In this scenario ethical approvals consider privacy of patients and also any potential impact on their management. All patients gave written consent for their CT images to be used for research. All centers had permission from their institutional review board (research ethics committee) to use existing CT data for this research on diagnostic accuracy of Computer Assisted Detection on the condition that data were made anonymous. All radiologists signed written clinical trial agreements with the company running these trials (Medicsight Ltd., Hammersmith, UK) to participate in research re-interpreting CT colonography images for studies of diagnostic accuracy with and without Computer Assisted Detection software provided by Medicsight Ltd.

The first study investigated 10 radiologists with no prior experience of CTC who interpreted 107 patients unaided and with concurrent CAD [8]. Although inexperienced with CTC, the readers in this study were fully trained radiologists (defined by having passed the Fellowship of the Royal College of Radiologists of the United Kingdom and having achieved the Certificate of Completion of Specialist Training, which is equivalent to board certification). They read a review article to familiarise themselves with CTC and principles of interpretation and were aware that the study aimed to evaluate CAD for CTC. They were unaware of the prevalence of abnormality in the dataset other than being told that some patients had normal findings and some had abnormal findings and that the CAD system could make both true-positive and false-positive marks, and could also miss polyps.

The second study investigated 16 experienced radiologists (mean 264 CTC cases interpreted prior to the study, individual numbers as follows: 50, 50, 90, 90, 100, 120, 130, 150, 200, 200, 250, 300, 500, 500, 500, >1000). These readers interpreted 112 patients unaided and with both concurrent and second-read CAD [10]. Again they were unaware of the prevalence of abnormality in the dataset.

### Data characteristics

118 different patients were interpreted prospectively across the two studies, with 102 patients common to both. These 102 cases therefore enabled paired comparisons of experienced and inexperienced observers without the need for imputation to account for missing data; i.e. differences could be attributed directly to experience rather than case mix. We calculated per-case differences between novices and experienced readers, thereby allowing data clustering to be included in the analysis, generating more appropriate 95% confidence intervals. Cases were both symptomatic and asymptomatic, and were aggregated from three USA and two European centres who had obtained ethical approval for anonymised patient data sharing. Prone and supine CTC used multidetector-row machines following full bowel purgation, adhering to contemporaneous guidelines [14].

A ground-truth reference standard against which to judge CAD and reader output was established by three experienced radiologist readers (>200 endoscopically validated cases) for each patient case. None of these radiologists were study readers. A pair read each case with the original radiological, endoscopic and histological reports available, reaching consensus for case classification and the size/location of any polyp encountered: 46 cases were judged normal and 56 had at least one polyp. There were 132 polyps in total: 15 ≥10mm, 41 6–9mm, 76 ≤5mm, with 12, 25 and 19 cases respectively where these were the largest polyps. In 37 cases the largest polyp was at least 6mm.

### Reading environment and CAD paradigm

Inexperienced readers interpreted cases without CAD in a quiet environment over one week, repeating interpretation with concurrent CAD [15] two months later to diminish the effect of recall bias. Experienced readers interpreted cases in three batches, each over one month, with at least one month between batches to diminish recall bias [10]. All cases were read once in each batch, using one of three paradigms (unassisted, concurrent-CAD, second-read CAD), with paradigm and case sequence randomised between batches. Thus unassisted interpretation and concurrent-CAD were common to both studies, with a temporal separation of at least one month between the same case. For the concurrent paradigm, readers interpreted CAD-annotated CTC data simultaneously with unannotated data [16]. Because the same CAD system algorithm was used for both studies (Colon CAD API 3.1, Medicsight, Hammersmith, UK), true-positive and false-positive detections were identical for individual patients used across the studies, eliminating this as a confounder. A proprietary CTC viewing package was used by inexperienced readers while experienced readers used CAD in a commercial implementation (either Viatronix V3D, Stony Brook NY, USA, or Vital Images, Minnetonka, Minn, USA). Readers in both studies received comprehensive training regarding how to use the CAD system [8, 10]. No cases had been used previously for development of the CAD algorithm.

For each patient case, readers indicated whether they believed a polyp present or not, irrespective of its size. They indicated the segmental location and coordinates of any polyps detected, and maximum diameter using software callipers. Responses were made on study datasheets collated subsequently by a study coordinator.

### Statistical analysis

Via comparison to the ground truth reference for each case, readers’ responses were classified as true-positive, true-negative, false-positive, false-negative for each individual patient. Individual polyps were also categorised as true-positive or false-positive. The raw data for the study are provided in the Appendix (S1 Appendix).

We anticipated that CAD assistance would increase diagnostic sensitivity while simultaneously decreasing specificity. Because it has been established that gains in sensitivity are clinically more desirable than corresponding loss of specificity in the context of colorectal cancer screening [17], our pre-specified primary outcome was a net benefit measure that combined sensitivity and specificity as a single metric weighted in favour of sensitivity for detection of patients with any polyp [18, 19]. The net benefit of CAD assistance was defined as:

CAD net benefit=[Δsensitivity+Δspecificity×(1/W)×(1−p)/p]

Δsensitivity was the change in sensitivity, Δspecificity the change in specificity from baseline (i.e. without CAD) for detection of patients with any polyp, achieved with CAD [18], and p was the proportion of patients with polyps in the intended population (i.e. asymptomatic screenees); 25%. W was derived from a prior discrete choice experiment that determined the value patients and healthcare professionals placed on enhanced sensitivity over and above correspondingly diminished specificity in the context of colorectal cancer screening [17]: The average participant regarded one additional true-positive polyp equivalent to 6 additional false-positives. [17];

Net benefit overall was calculated by meta-analysis, treating each reader as if an individual study. Average estimates were calculated from bootstrap samples generated by random sampling of patient cases for each reader, bootstrapping positive and negative patients separately. Confidence intervals (CI) were calculated from the 2.5% and 97.5% percentiles of the cumulative distribution of bootstrap estimates. We defined a significant benefit for CAD as a positive net benefit whose 95% confidence interval excluded zero.

The following secondary outcomes were pre-specified for experienced and inexperienced readers, and the difference between them:

- Per-patient sensitivity and specificity unassisted, with concurrent CAD, and the change with CAD, both for patients with any polyp and patients with polyps ≥6mm
- Per-polyp sensitivity unassisted, when using CAD, and the change with CAD, for patients with any polyp, polyps ≥6mm, and polyps ≤5mm.
- The mean number of patients correctly classified as true-positive solely due to false-positive detections.
- Mean reading time with and without CAD, and the difference.

We wished to speculate on the potential gain for inexperienced readers when using second-read CAD by quantifying the difference in accuracy between concurrent and second-read paradigms for experienced readers via existing data.

Average estimates were calculated from 2000 bootstrap samples via random sampling of patients and readers, retaining data clustering. Positive and negative patients were bootstrapped separately and the same bootstrap samples of cases used for both studies. Readers were bootstrapped separately for each study. Differences between inexperienced and experienced readers were calculated within each case then averaged across all cases. Meta-analysis with equal weighting per reader was used to obtain an overall average. Per-polyp sensitivity analysis accounted for clustering of multiple polyps per patient. Confidence intervals were calculated from the 2.5% and 97.5% percentiles of the cumulative distribution of the 2000 estimates. Although underpowered for analysis at the 1cm threshold, we calculated the median number of patients detected here. Interpretation times for experienced readers were based on 15 readers (one had missing data). Sensitivity and specificity, and changes in these were expressed as percentages with 95% confidence intervals (CI). Confidence intervals excluding zero were considered statistically significant.

## Results

### Per-patient analyses

CAD changed the proportion of both inexperienced and experienced correctly identifying cases with polyps in 83% of cases; detection of patients with polyps increased in 70% and 57% of cases over 10 and 16 readers respectively. Per-patient sensitivity and specificity (with 95%CI) with and without CAD are shown in Table 1. There was significant gain in sensitivity with CAD for all polyps of 14.1% for inexperienced readers (rising from 39.1% to 53.2%). Sensitivity for patients with any polyp was higher for experienced readers but the mean gain of 4.6% with CAD was not significant (rising from 57.5% to 62.1%).

**Table 1:** Per-patient results for net benefit of CAD assistance when used in concurrent mode for interpretation of CT colonography by inexperienced and experienced readers. For all comparisons differences are calculated as performance with CAD assistance minus performance when unassisted. All data are percentages.

	Inexperienced readers [mean % (95%CI)]	Experienced readers [mean % (95%CI)]	Difference: Inexperienced–Experienced [mean % (95%CI)]
CAD net benefit measure (all polyps)	11.2 (3.1 to 18.9)	3.2 (-1.9 to 8.3)	7.9 (-0.9 to 16.6)
Unassisted sensitivity (all polyps)	39.1 (30.9 to 47.0)	57.5 (49.6 to 65.2)	-18.5 (-25.3 to -11.9)
Unassisted specificity (all polyps)	94.1 (90.0 to 97.4)	91.0 (87.0 to 94.8)	3.1 (-1.7 to 7.9)
Sensitivity with CAD (all polyps)	53.2 (43.9 to 61.4)	62.1 (54.1 to 70.3)	-8.9 (-16.6 to -1.9)
Specificity with CAD (all polyps)	88.0 (82.2 to 93.3)	88.3 (83.8 to 92.4)	-0.3 (-5.6 to 5.0)
Change in sensitivity with CAD (all polyps)	14.1 (6.8 to 21.4)	4.6 (-0.2 to 9.3)	9.6 (1.2 to 17.7)
Change in specificity with CAD (all polyps)	-6.1 (-12.0 to -0.2)	-2.7 (-6.3 to 0.8)	-3.4 (-9.6 to 3.0)
CAD net benefit measure (polyps ≥6mm)	9.9 (0.1 to 19.2)	3.7 (-2.6 to 10.1)	6.1 (-4.0 to 15.6)
Unassisted sensitivity (polyps ≥6mm)	49.5 (40.0 to 58.9)	65.9 (56.4 to 74.7)	-16.4 (-24.0 to -8.3)
Unassisted specificity (polyps ≥6mm)	92.6 (89.0 to 95.5)	93.5 (90.5 to 95.9)	-0.9 (-4.4 to 2.5)
Sensitivity with CAD (polyps ≥6mm)	61.1 (50.0 to 71.1)	70.1 (60.5 to 78.7)	-9.0 (-18.4 to -0.3)
Specificity with CAD (polyps ≥6mm)	89.2 (84.7 to 92.8)	92.7 (89.0 to 95.5)	-3.5 (-7.4 to 0.2)
Change in sensitivity with CAD (polyps ≥6mm)	11.6 (1.9 to 20.5)	4.2 (-2.0 to 10.5)	7.5 (-2.6 to 16.8)
Change in specificity with CAD (polyps ≥6mm)	-3.4 (-8.0 to 0.8)	-0.8 (-3.4 to 1.7)	-2.6 (-7.5 to 2.0)

Inexperienced readers benefitted by a mean gain in sensitivity approximately three times greater than for experienced readers, a significant difference of 9.6% (95%CI 1.2% to 17.7%). The mean fall in specificity of -6.1% with CAD was significant for inexperienced readers (falling from 94.1% to 88.0%) whereas the mean fall of -2.7% with CAD was nonsignificant for experienced readers (falling from 91.0% to 88.3%). Thus, in 200 patients (100 with polyps) inexperienced readers using CAD would on average correctly identify 14 additional patients with polyps, at the cost of approximately 6 additional false-positives, whereas experienced readers would identify 4 or 5 additional patients with polyps at cost of 2 or 3 additional false-positives. For our primary outcome, these data gave a mean CAD net benefit of 11.2% (95%CI 3.1% to 18.9%) for inexperienced readers versus 3.2% (95%CI -1.9% to 8.3%) for experienced, with a mean difference of 7.9% (95%CI -0.9% to 16.6%) between the two groups (Table 1).

Restricting the analysis to patients with polyps ≥6mm found a significant mean rise in sensitivity with CAD of 11.6% for inexperienced readers (rising from 49.5% to 61.1%) versus 4.2% for experienced readers (rising from 65.9% to 70.1%), which was not significant (Table 1). In this case, however, the fall in specificity with CAD was non-significant for both groups, with a mean fall of 3.4% for inexperienced readers versus 0.8% for experienced. Thus, of 200 patients (100 with polyps) inexperienced readers using CAD would on average correctly identify 11 or 12 additional patients with polyps ≥6mm, at the cost of approximately 3 or 4 additional false-positives, whereas experienced readers would identify 4 or 5 additional patients at the cost of 1 additional false-positive. Net benefit was significant for inexperienced readers (9.9%, 95% CI 0.1% to 19.2%) but not experienced readers (3.7%, 95%CI -2.6% to 10.1%), with a non-significant difference between groups (6.1%, 95%CI -4.0% to 15.6%) (Table 1).

### Per-polyp analyses

Per-polyp sensitivities are shown in Table 2. For all polyps there was a significant mean rise in sensitivity with CAD of 9.0% for inexperienced readers (rising from 15.4% unassisted to 24.4%) compared with 4.1% for experienced readers (rising from 30.3% to 34.4%), also significant. Restricting analysis to polyps ≥6mm the mean rise of 10.0% (rising from 28.5% to 38.5%) for inexperienced readers was significant but the mean rise of 3.0% (rising from 51.0% to 54.0%) for experienced readers was not. With analysis restricted to polyps ≤5mm the mean rise in sensitivity with CAD was significant for both groups, 8.3% (rising from 5.9% to 14.2%) for inexperienced readers and 4.8% (15.3% rising to 20.1%) for experienced readers. The magnitude of benefit with CAD was not significantly different between the two groups.

**Table 2:** Per-polyp sensitivity for CAD assistance when used in concurrent mode for interpretation of CT colonography by inexperienced and experienced readers. For all comparisons differences are calculated as performance with CAD assistance minus performance when unassisted. All data are percentages.

	Novice readers [mean % (95%CI)]	Experienced readers [mean % (95%CI)]	Difference (Novice–Experienced)
Unassisted sensitivity (all polyps)	15.4 (11.3 to 20.8)	30.3 (23.9 to 37.7)	-14.9 (-19.6 to -10.5)
Sensitivity with CAD concurrent (all polyps)	24.4 (18.8 to 31.3)	34.4 (27.4 to 42.5)	-10.0 (-14.7 to -5.4)
Change in sensitivity with CAD (all polyps)	9.0 (5.1 to 13.2)	4.1 (1.0 to 7.5)	4.9 (0.3 to 9.5)
Unassisted sensitivity (polyps ≥6mm)	28.5 (20.2 to 36.9)	51.0 (40.4 to 60.9)	-22.5(-29.9 to -14.7)
Sensitivity with CAD (polyps ≥6mm)	38.5 (29.7 to 48.3)	54.0 (43.0 to 64.7)	-15.5 (-23.0 to -7.6)
Change in sensitivity with CAD (polyps ≥6mm)	10.0 (3.0 to 17.3)	3.0 (-2.1 to 8.7)	7.0 (-1.2 to 14.7)
Unassisted sensitivity (polyps ≤5mm)	5.9 (3.0 to 10.0)	15.3 (10.4 to 21.2)	-9.3 (-14.3 to -5.6)
Sensitivity with CAD (polyps ≤5mm)	14.2 (8.4 to 21.4)	20.1 (13.9 to 28.0)	-5.9 (-10.6 to -0.9)
Change in sensitivity with CAD (polyps ≤5mm)	8.3 (4.1 to 13.6)	4.8 (1.5 to 8.7)	3.5 (-1.2 to 8.9)

### Second-read CAD

Data for second-read CAD were only available for experienced readers and are shown in Table 3 with 95%CI. There was a significant rise in mean sensitivity of 6.9% for patients with all polyps (rising from 57.5% to 64.4%), with a non-significant fall in mean specificity of -2.0% (falling from 91.0% to 89.0%). Thus of 200 patients (100 with polyps) experienced readers would identify 6 or 7 additional patients with polyps on average, at a cost of 2 additional false-positives. These data gave a significant CAD net-benefit of 6.0 (95%CI 1.2 to 10.5). Mean per-patient sensitivity for patients with polyps ≥6mm rose significantly by 6.9% also, with a non-significant fall in specificity of -0.9%. Per-polyp sensitivity rose significantly by a mean of 7.2% for all polyps, with significant gains in mean sensitivity of 9.1% for polyps ≥6mm and 5.8% for polyps ≤5mm.

**Table 3:** Effect of CAD assistance when used in second-read mode for interpretation of CT colonography by experienced readers. For all comparisons differences are calculated as performance with CAD assistance minus performance when unassisted. All data are percentages.

	Per-Patient analysis [mean % (95%CI)]	Per-polyp analysis [mean % (95%CI)]
CAD net benefit measure (all polyps)	6.0 (1.2 to 10.5)	n/a
Unassisted sensitivity (all polyps)	57.5 (49.6 to 65.2)	30.3 (23.9 to 37.7)
Unassisted specificity (all polyps)	91.0 (87.0 to 94.8)	n/a
Sensitivity with CAD (all polyps)	64.4 (56.6 to 72.3)	37.5 (29.5 to 46.1)
Specificity with CAD (all polyps)	89.0 (84.1 to 93.3)	n/a
Change in sensitivity with CAD (all polyps)	6.9 (2.8 to 11.2)	7.2 (3.9 to 10.6)
Change in specificity with CAD (all polyps)	-2.0 (-6.2 to 1.6)	n/a
CAD net benefit measure (polyps ≥6mm)	6.6 (1.2 to 11.9)	n/a
Unassisted sensitivity (polyps ≥6mm)	65.9 (56.4 to 74.7)	51.0 (40.4 to 60.9)
Unassisted specificity (polyps ≥6mm)	93.5 (90.5 to 95.9)	n/a
Sensitivity with CAD (polyps ≥6mm)	72.8 (63.3 to 81.4)	60.1 (48.9 to 70.4)
Specificity with CAD (polyps ≥6mm)	92.6 (89.0 to 95.6)	n/a
Change in sensitivity with CAD (polyps ≥6mm)	6.9 (1.9 to 12.5)	9.1 (3.8 to 13.8)
Change in specificity with CAD (polyps ≥6mm)	-0.9 (-3.7 to 1.8)	n/a
Unassisted sensitivity (polyps ≤5mm)	n/a	15.3 (10.4 to 21.2)
Sensitivity with CAD (polyps ≤5mm)	n/a	21.1 (14.3 to 29.7)
Change in sensitivity with CAD (polyps ≤5mm)	n/a	5.8 (2.3 to 9.7)

Second-read CAD was not tested with inexperienced readers but we can expect at least a similar impact to that seen in experienced readers, indeed likely greater. Using second-read CAD experienced readers achieved an average sensitivity 2.5% greater than when using concurrent CAD (6.9% increase with second-read, 4.6% increase with concurrent read; Table 1 and Table 3). Specificity for experienced readers rose by 0.7% using second-read CAD compared to concurrent (-2.0% change for second-read versus -2.7% for concurrent; Table 1 and Table 3). Conservative estimates suggest a significant rise in sensitivity for inexperienced readers of 16.6% (14.1% plus 2.5%; Table 1) with a potentially significant fall in specificity of approximately -5.5% (-6.1% plus +0.7%; Table 1).

### Other analyses

It is possible to fortuitously achieve a true-positive patient diagnosis by assigning a false-positive polyp while simultaneously missing a true polyp. The mean number of such occasions was 4.3 for both experienced and inexperienced readers when unassisted, falling with CAD to 3.9 for experienced readers and rising to 5.0 for inexperienced readers. Thus the proportion of such patients was small overall and the effect on analysis was negligible; i.e. increased sensitivity with CAD was not due to false-positive detections in patients with true polyps elsewhere.

When unassisted, mean reading time for inexperienced readers was 11.2 min (95%CI 10.7 to 11.7) compared with 7.9 min (7.4 to 8.2) for experienced readers. When using CAD concurrently, this fell to 8.9 (8.3 to 9.4) for inexperienced readers but rose to 8.7 (8.2 to 9.3) for experienced readers.

## Discussion

We aimed to quantify the incremental benefit of CAD for inexperienced versus experienced readers; both groups read the same CTC data using concurrent CAD and the same CAD algorithm. Our primary outcome was the net benefit when using CAD to detect patients with polyps of any size [19, 20]. Inexperienced readers achieved a significant net benefit of 11.2% with CAD but experienced readers only achieved 3.2%; i.e. net benefit for inexperienced readers was nearly four times greater. This occurred despite a significant fall in specificity with CAD for inexperienced readers (a phenomenon not seen with experienced readers), i.e. in our weighted analysis raised sensitivity outweighed simultaneously diminished specificity. For both groups the impact of CAD was spread across 83% of cases with polyps, indicating that benefit was not confined to a small number of pivotal, individual cases. When the analysis was restricted to patients with polyps ≥6mm we again found that net benefit was greatest for inexperienced readers. Per-polyp analyses found that inexperienced readers achieved gains in sensitivity when CAD-assisted for polyps of all sizes and also when restricted to polyps ≥6mm and ≤5mm. Experienced readers also achieved significant gains in sensitivity for the “all polyps” and “≤5mm” analyses but not polyps ≥6mm.

Several studies have investigated CAD-assistance for inexperienced readers since it is believed that diagnostic gains will be maximised in this group. However, direct comparisons with experienced readers are uncommon, possibly because experienced readers are difficult to recruit versus inexperienced readers, who are often trainees and/or those wishing to learn CTC. Mang [6], found second-read CAD raised sensitivity for two inexperienced readers to levels close to that achieved by two experienced readers. Our findings suggest that while CAD improves sensitivity for inexperienced readers, it cannot do so enough to compensate for a lack of proper training and experience. Supporting this, a study of 6 inexperienced participants from a prior CAD study found that a single day of clinical training significantly increased subsequent sensitivity [21]. Researchers have investigated CAD for training inexperienced readers[22].

Second-read CAD was restricted to experienced readers, in whom net benefit was found to be greater than for concurrent CAD, suggesting second-read is the more diagnostically accurate paradigm. Other researchers have also found second-read CAD beneficial for experienced readers [9]. We did not test second-read CAD on inexperienced readers but it is plausible to expect at least a similar benefit to that observed in experienced readers, with a similar magnitude of difference between paradigms. In reality, a larger net benefit is likely for inexperienced readers since they achieved proportionally more benefit from concurrent CAD than did experienced readers. We assume that second-read CAD would increase sensitivity for patients with any polyp by approximately 16.6% while decreasing specificity by approximately -5.5%. A study of seven “moderately” experienced readers (40 to 150 previous cases) found that both CAD paradigms improved diagnostic performance significantly, especially concurrent [12].

Our primary outcome involved patients found to have polyps of any diameter. All such patients are potential candidates for colonoscopy since there is disagreement between clinicians regarding the appropriate polyp diameter for referral [23]. Indeed, three or more diminutive polyps alone attract higher CRADS scores [24]. Since small polyps are most difficult to detect, we would expect CAD assistance to exert most impact here.

Our analysis accounted for unequal misclassification costs [18–20]; in clinical terms, a false-positive diagnosis entails unnecessary colonoscopy whereas a false-negative means a missed polyp and therefore potentially a missed cancer. We believe that a major benefit of the present study is that our analysis used a weighting that had been obtained from a discrete choice experiment designed and executed specifically to obtain a precise value for this weighting [17]; prior analysis of a portion of these study data has used a more conservative weighting factors of three, derived by expert opinion [8]. Giving the views of patients and healthcare professionals equal precedence resulted in a weighting of six in favour of sensitivity. This factor rose to 22 for patients alone and to 2250 for cancer versus polyps [17]. These data are similar to those observed in mammographic screening where women will trade an average of 500 false-positive diagnoses in return for a single additional cancer [25]. It could be argued we should have used a weighting factor greater than six, since this applies to polyps only. Instead our weighting could have accounted for the fact that cancers are also detected when screening, albeit much less frequently than adenomatous polyps.

Unassisted interpretation time was longest for inexperienced readers. Although we might expect experienced readers to be quicker, it could be argued that optimal diagnostic accuracy arises from slow, careful inspection. Concurrent CAD shortened interpretation for inexperienced readers but raised it for experienced readers. Inexperienced readers may have given less attention to un-annotated colon, suggesting “over-reliance” on CAD. Experienced readers may be more wary of CAD (although both groups were told that CAD could be inaccurate). Recent paradigms incorporating an initial first-read with CAD have been found time-efficient compared to second-read paradigms [26].

Our study has limitations. Inexperienced participants read under “laboratory” conditions over a week whereas experienced readers’ read over a month at work, which may have exerted an influence. The CAD algorithm was identical and so had identical true-positive and false-positive marks for the same case, but display platform differed: inexperienced readers used an in-house interface whereas experienced used commercially-available workstations. However, the types of display were similar (i.e. including axial prone and supine views, and 3D rendering) and we would expect any differences to be dwarfed by the differences encountered when using CAD assistance. Our assumption that second-read CAD would benefit inexperienced readers is based on direct comparison between groups using concurrent CAD coupled with the incremental benefit of second-read over concurrent for experienced readers, which is statistically plausible but speculative. While the second read paradigm is most often stipulated for the licencing of CAD systems, other paradigms (such as the concurrent paradigm investigated in the current paper) have received research attention. Indeed, recent work has focussed on a “first-read” paradigm where CAD is deployed first rather than last, with investigators finding it as effective as the second-read paradigm while being more time efficient [26]. Our experienced readers had a median experience of 264 cases prior to the study. Fifty cases has historically been regarded as the number necessary to gain competence but this figure was based on expert consensus. A study of novice readers found that interpretation of 164 cases was necessary in order to reach the sensitivity obtained by experienced readers (who were defined by prior interpretation of 350 cases) [27]. It is therefore possible that eight of our “experienced” readers were not at this level. The difference, if any, between “experienced” (i.e. competent) and “expert” readers still requires definition.

In summary, concurrent CAD conveyed significant net benefit for inexperienced readers when identifying patients with any polyp. Benefit was approximately four times the magnitude observed in experienced readers. Experienced readers found second-read CAD more beneficial than concurrent, suggesting it would also be more effective for inexperienced readers.

## Supporting Information

S1 Appendix Raw data file. (XLS)
